# Island method for estimating the statistical significance of profile-profile alignment scores

**DOI:** 10.1186/1471-2105-10-112

**Published:** 2009-04-20

**Authors:** Aleksandar Poleksic

**Affiliations:** 1 Department of Computer Science, University of Northern Iowa, Cedar Falls, IA 50614, USA

## Abstract

**Background:**

In the last decade, a significant improvement in detecting remote similarity between protein sequences has been made by utilizing alignment profiles in place of amino-acid strings. Unfortunately, no analytical theory is available for estimating the significance of a gapped alignment of two profiles. Many experiments suggest that the distribution of local profile-profile alignment scores is of the Gumbel form. However, estimating distribution parameters by random simulations turns out to be computationally very expensive.

**Results:**

We demonstrate that the background distribution of profile-profile alignment scores heavily depends on profiles' composition and thus the distribution parameters must be estimated independently, for each pair of profiles of interest. We also show that accurate estimates of statistical parameters can be obtained using the "island statistics" for profile-profile alignments.

**Conclusion:**

The island statistics can be generalized to profile-profile alignments to provide an efficient method for the alignment score normalization. Since multiple island scores can be extracted from a single comparison of two profiles, the island method has a clear speed advantage over the direct shuffling method for comparable accuracy in parameter estimates.

## Background

The statistical significance of a local alignment score between two sequences of amino-acid letters can be assessed by analyzing background distribution of the alignment scores between random sequences. For Smith-Waterman alignments [1] lacking gaps, it has been well established that the background score distribution is approximately Gumbel [2], specified by two analytically computable parameters *λ *and *K *[3-6].

Assessing score statistics for profile-based alignments is much more challenging problem. In order to quickly estimate the significance of a database match, the HMMER method (Eddy, 1997) pre-computes extreme value distribution parameters for each Hidden Markov model in the profile library. These model dependent parameters are calculated by aligning and scoring a given HMM against thousands of real or random sequences. PSI-BLAST estimates score significance "on the fly", by reconstructing residue scores within each profile column to the same scale as the scores specified in the BLOSUM62 matrix [7]. The assumption is that, after rescaling, the background distribution of PSI-BLAST scores will be the same as the distribution of the gapped BLAST scores. Many experiments suggest that this hypothesis is valid and that the rescaling technique yields accurate *p*-values.

The assessment of statistical significance of profile-profile scores is still an unsolved problem. In lieu of a rigorous analytical theory, many profile-profile algorithms resort to Z-score statistics [8,9]. For sequence only methods, the Z-value of an alignment score between two sequences is computed by comparing the first sequence with randomly shuffled versions of the second sequence. An advantage of Z-values is that they eliminate the sequence length and compositional bias, since the shuffling of a sequence preserves these two variables. However, there are certain disadvantages to using raw Z-scores to rank the significance of the alignment scores. First, the Z-score statistics makes a false assumption about the Gaussian form of the underlying score distribution. A reader interested in the magnitude of the error introduced by this assumption in referred to [10]. Second, Z-scores do not provide the probability that an alignment score could be obtained by chance.

Nevertheless, the Z-values can be made very useful for computing accurate p-values via a "change of variable" technique [11]. More specifically, it has been shown that if the raw alignment scores follow a standard Gumbel law, then the *p*-values of associated Z-scores are free of sequence length and amino acid composition biases [12,13]. Since the only drawback of this approach is the computational expense associated with random simulations, it would be very interesting to see whether the "change of variable" approach can be used in other settings.

Recently, an interesting approach to alignment score normalization has been described that uses so-called Shared Amount of Information (SAI) between the amino-acid[12]. The model proposed in [12] is unique since it is derived from the reliability theory applied to sequences of amino-acids.

To date the studies on score normalization for local profile-profile alignments have been limited to some specific alignment scoring schemes. For example, an explicit generalization of techniques implemented in PSI-BLAST has been successfully used in the COMPASS algorithm [14]. However, the method described in Sadreyev *et al*. works only in the context of the COMPASS scoring function. The statistical significance of alignment scores produced by the LAMA method is estimated using an approach based on Fisher's combining method [15]. In HHSEARCH [16], the profile specific parameters were computed by comparing each profile to the set of profiles built for the representative sequences in the SCOP database [17] (SCOP folds). The alignment scores obtained by PROF_SIM [18], STRUCTFAST[19], and UNI-FOLD [20] were also shown to follow the extreme value distribution, but the distribution parameters in these methods must be pre-calculated using computationally expensive curve-fitting procedure. This approach is commonly referred to as the "direct method". In the "direct method", thousands of optimal alignment scores between real or random profiles are usually needed for moderately accurate estimates of the distribution parameters. On the other hand, profile-profile methods are computationally very expensive, making the direct method too slow for parameter estimation, in particular for deriving the score statistics "on the fly" for each given pair of profiles.

Here, we study a generalization of the well known island method [21,22] to score normalization problem for profile-profile alignments. The island method uses the scores of local alignment "islands" obtained by a simple modification of the dynamic programming matrix. Since multiple island scores can be computed from a single path graph, the island method has a distinct speed advantage over the direct method.

## Methods

### The statistical theory

The statistical significance of an alignment score is usually expressed by the score's *p-*value. The *p-*value of a score *x *is defined as the probability of obtaining a score of at least *x *purely by chance, given the probabilistic models for the sequences and the alignment scoring scheme.

For a pair of random sequences of lengths *m *and *n*, the expected number of locally optimal gapless sub-alignments with score of at least *x *is approximately Poisson distributed with mean value E given by

(1)

The analytically computable parameters *λ *and *K *depend on the background probabilities of amino-acid letters and the residue-residue substitution scores specified in the mutation matrix.

The equation 1 implies that the *p-*value of a score *x *is

(2)

There is plenty of evidence suggesting that equation 1 still holds for alignments with gaps [23-28], as well as for profile-sequence and profile-profile alignments[7,18,29]. However, for these methods, *λ *and *K *must be estimated from random simulations rather than computed analytically [3,18,28,8]. We note that precise estimates of *λ *are particularly important since the *p*-value is a doubly exponential function of *λ*. We also note that, in contrast to local alignment scores, the scores of global sequence-sequence alignments are shown to approximately follow a three-parameter gamma distribution function[31]. For global alignment statistics, the computational complexity is still an open problem.

### Need for composition-based statistics for profile-profile alignments

For alignment methods that use substitution matrices and residue type information (such as BLAST[4] or FASTA[32]), it has been well established that *λ *and *K *depend, not only upon the alignment scoring system, but also upon the frequencies of amino-acid letters in the sequences being aligned. In these methods, *λ *can vary more than 10% from one sequence pair to another, due entirely to change in sequence amino-acid composition [21].

**Figure 1:**
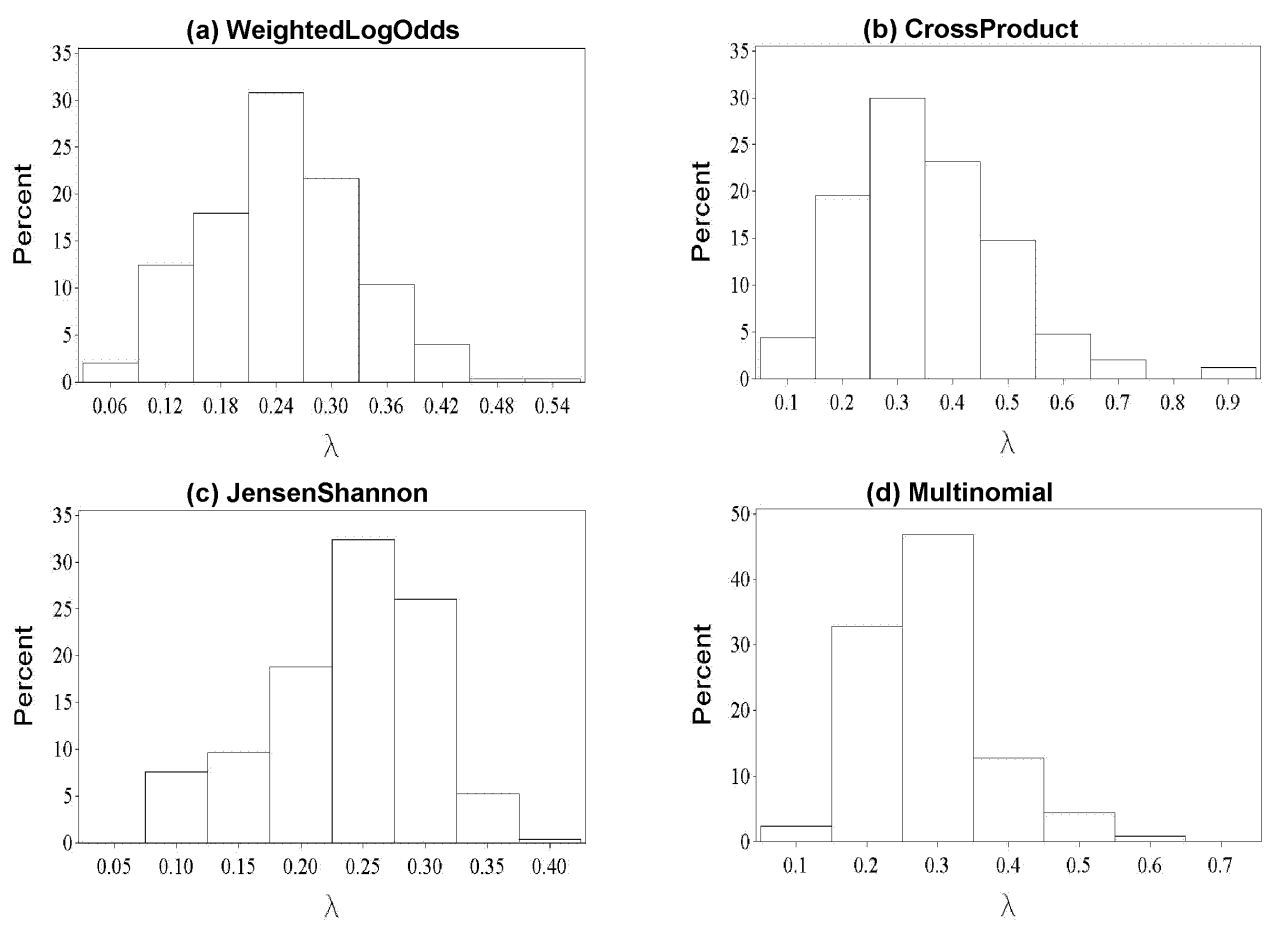
**Profile-pair specific estimates of *λ***. The histogram of estimates of *λ *for 500 pairs of profiles selected at random from the set of profiles built for representative sequences in the FSSP database. For each pair of profiles, the distribution parameters were fit to 10,000 optimal alignment scores between the profiles' shuffles. The standard error in each estimate of *λ *is 0.78%. The mean and standard deviation of *λ *are: (a) μ = 0.244, σ = 0.093 (b) μ = 0.353, σ = 0.139 (c) 0.238, σ = 0.067 (d) μ = 0.283 σ = 0.089. For sequence only comparisons, μ = 0.307 and σ = 0.048.

**Figure 2:**
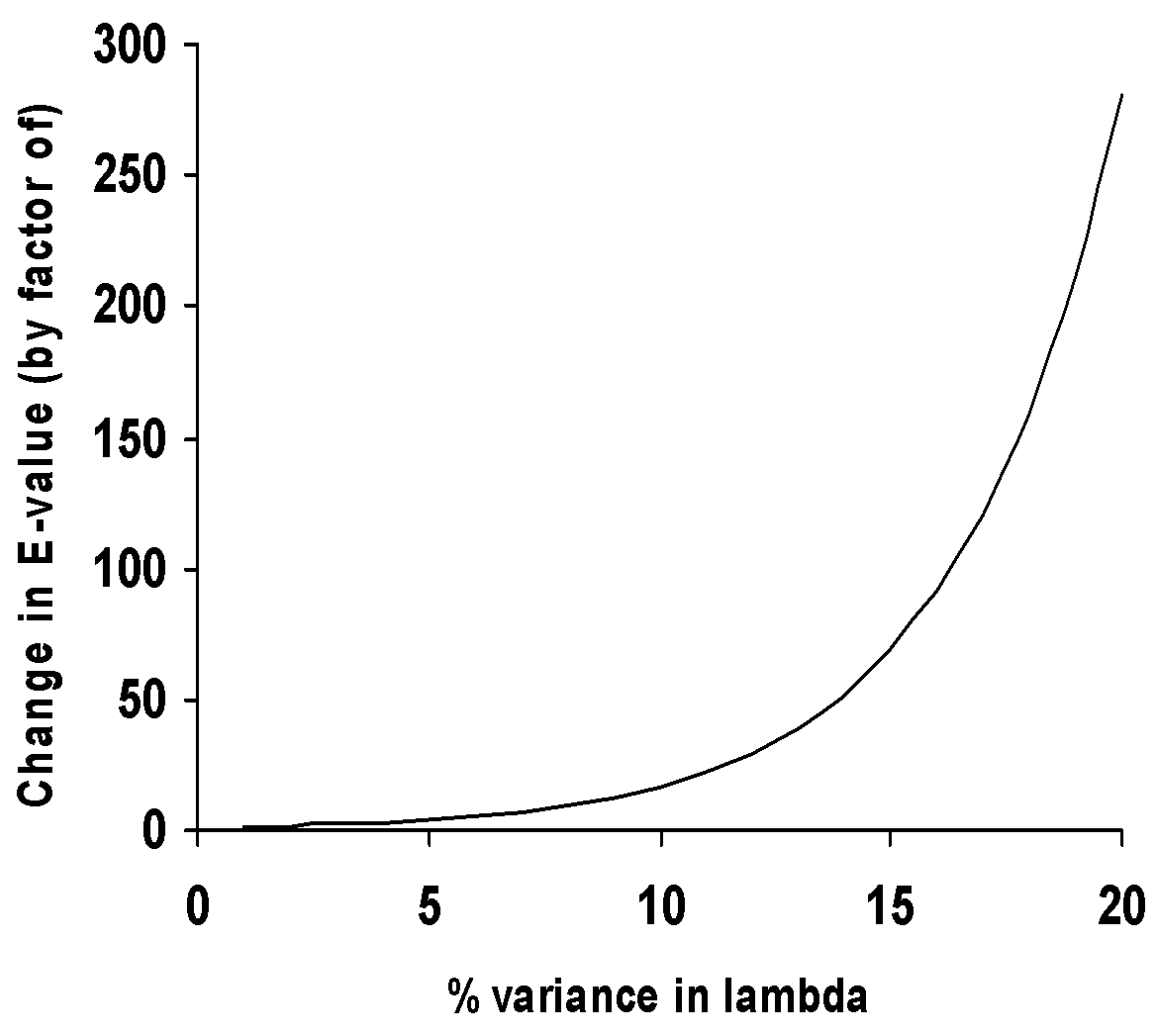
**Change in E-value as a function of variance in *λ***. Impact of variance in *λ *on E-values for marginally significant alignment scores (*p*-value ≈ 10^-9^) between profiles of lengths 350. For example, 1%, 3%, and 5% error in lambda leads to an error in E-value by a factor greater than 1.3, 2.3, and 4, respectively. On the other hand, 20% change in *λ *leads to an almost 300 fold change in the estimated E-value.

The variation in *λ *is much larger for profile-profile methods. Figure 1 shows the histogram of estimates of *λ *for 500 pairs of profiles selected at random from the set of profiles constructed for representative sequences in the FSSP database [33]. For each pair of profiles, *λ *is computed by repeatedly shuffling the columns (positions) in both profiles and fitting statistical parameters to optimal local alignment scores between profiles' shuffles. As seen in Figure 1, for some alignment methods, the difference in *λ *between pairs of profiles reaches an order of magnitude. On the other hand, for marginally significant alignment scores between average length profiles, even a relatively small change in *λ *of 10% results in over 16 fold change in the estimated E-value (see Figure 2). This implies that E-values computed for profile-profile scores using any fixed *λ *are unreliable, establishing a need for computing the statistical parameters independently, for each given pair of profiles.

### Island statistics

To circumvent the computational expense associated with random simulations for sequence-sequence methods, Olsen *et al*. proposed using the scores of the so-called "alignment islands" [22]. An alignment island is a region in the dynamic programming matrix corresponding to positively scoring segments in two sequences. More precisely, an island is a collection of locally optimal alignments that start at the same cell (anchor cell) in the path graph [21,22]. The score of an island is defined as the highest score among all local alignment scores for that island.

Since the accuracy of equation 1 increases with increasing values of *x*, accurate estimates of *λ *and *K *can be obtained by considering islands *i *with sufficiently high peak scores *σ *(*i*). Assuming continuity of alignment scores, the maximum likelihood estimate of *λ *is

(3)

where *R*_*c *_denotes the set of islands *i *such that *σ *(*i*) ≥ *c*[21]. The standard error in  is , where *λ *denotes the asymptotic parameter ("true" value). The maximum likelihood estimate of *K *is

(4)

where *m *and *n *are the lengths of the random sequences used in each island comparison and *B *is the total number of sequence comparisons performed to generate the islands[21].

We note that the island method is similar to the "declumping" method of Waterman and Vingron[26,27], but is much faster, because, unlike clumps, the islands and their scores can be collected with a minor modification of the Smith-Waterman algorithm [22]. Several applications have recently been developed that incorporate island statistics for score normalization, including CTX-BLAST [34], ConSequenceS[35], and CIS [36].

An added benefit of the island statistics (and other score normalization methods based on sequence shuffling) is flexibility in choosing the scoring system. In order to be amenable to island statistics, the only requirement a method needs to satisfy is that that the alignments it generates stay in the local regime, i.e. that the distribution of alignment scores between random sequences (profiles) is approximately Gumbel. Therefore, since the procedure for computing statistical parameters does not change with changes to the scoring function, one can entirely focus on improvements to the scoring scheme. This is important, because incorporating additional information into the alignment process, such as, for example, the compositionally adjusted background frequencies [20,37,38] or protein secondary structure information [9,39] is known to significantly increase sensitivity of an alignment method[9,16].

## Results and discussion

### The island statistics for profile-profile alignments

The alignment score significance can be assessed using either real or random profiles [40]. We use random profiles to avoid bias in the results toward any particular group of proteins. A random profile of length *n *is obtained by sampling *n *profile columns at random from the collection of profiles computed for ~2,500 representative sequences from the FSSP database (FSSP family representatives). The database of FSSP profiles is generated by running three PSI-BLAST iterations on each FSSP sequence and parsing amino-acid letter frequencies from the corresponding PSI-BLAST checkpoint files.

We study the applicability of the island statistics on four popular and well tested profile-profile scoring schemes: *JensenShannon *(implemented in the PROF_SIM method [18]), *CrossProduct *(PRALINE [39]), *WeightedLogOdds *(COMPASS[14]), and *Multinomial *(UNI-FOLD[20]). The definition of each scoring function is given in the appendix. The column-column scores in all four methods are scaled (multiplied by constant factors) so that the alignment score distributions have similar parameters.

**Figure 3:**
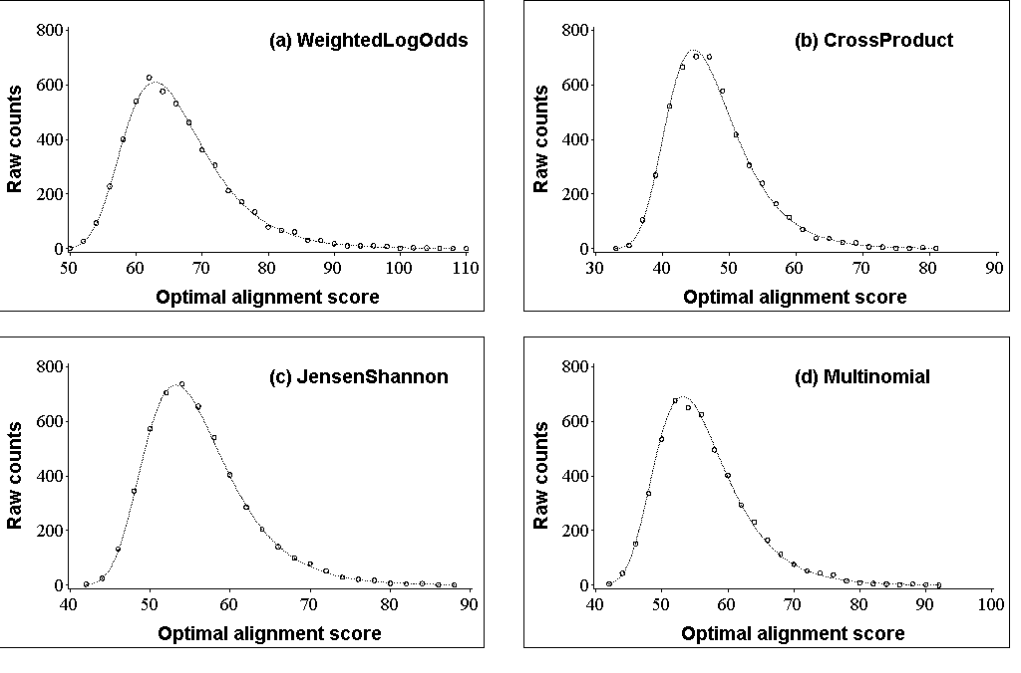
**Optimal alignment score distribution**. The distribution of 10,000 optimal local alignment scores between the shuffles of random profiles of lengths 1,500. Solid line represents the best-fit extreme value distribution. (a) *WeightedLogOdds: *A χ^2 ^goodness-of-fit test with 43 degrees of freedom has value 35.46, corresponding to a *P*-value of 0.79 (b) *CrossProduct*: *df *= 37, χ^2 ^= 32.47, *P*-value = 0.68 (c) *JensenShannon*: *df *= 39, χ^2 ^= 25.38, *P*-value = 0.95 (d) *Multinomial*: *df *= 39, χ^2 ^= 48.0, *P*-value = 0.15.

Since the island statistics applies only to methods for which the background distribution of optimal alignment scores is approximately Gumbel, we first verify that the algorithms in our study belong to this category. Figure 3 shows the score distributions of (globally) optimal local alignments between the shuffles of random profiles. As seen in Figure 3, for all four profile-profile methods in our study, the best-fit extreme value distribution closely follows the data, with *χ*^2 ^goodness-of-fit *p*-values ranging from 0.15 to 0.95.

To establish a link between the statistics of peak island scores and optimal alignment scores, we compare, for a range of cutoff values *c*, the observed number of islands with scores ≥ *c *with the expected number of such islands computed from the best-fit extreme-value distribution. The expected number of islands is defined as , where  and  are parameters obtained with the direct method. More specifically,  and  are the maximum likelihood estimates of parameters in equation 2, obtained from the scores of (globally) optimal local alignments between profile shuffles. For more on the maximum likelihood estimates of statistical parameters, the reader is referred to [41].

**Figure 4:**
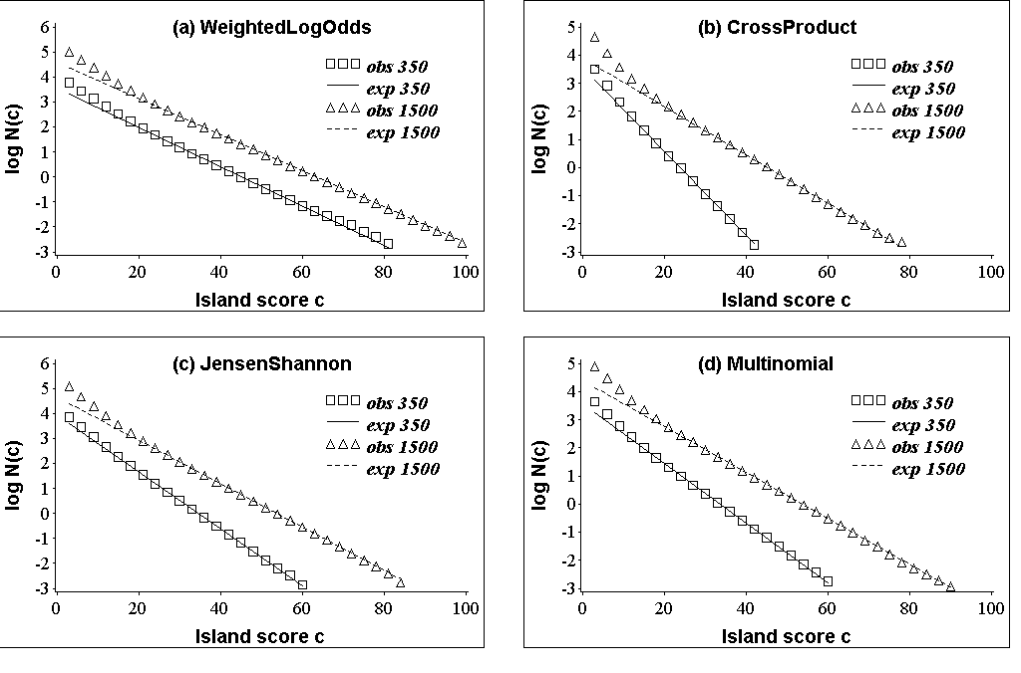
**Observed and expected island counts**. Semi-log plot of the observed and expected number of islands (per alignment) with score ≥ *c*. The islands were collected from 10,000 comparisons between the shuffles of random profiles.

As seen in Figure 4, there is strong agreement in the expected and observed counts of the island peak scores beyond the small score regime, independent of the scoring system employed and the lengths of the profiles. An analysis of real (as opposed to random) profiles demonstrates an equally strong correlation between two statistics for high scoring islands.

The two statistics obviously differ for low scoring islands (Figure 4). As argued before [21,22] the low scoring islands often correspond to ungapped alignments of only few profile positions, and therefore, the scores of those islands follow a different distribution, namely the distribution of gapless alignment scores.

The plots in Figure 4 show faster decay in the number of islands with score ≥ *c *for profiles of size 350 compared to profiles of size 1500 × 1500. We note that the apparent *λ *for each comparison in Figure 4 is equal to -*k*, where *k *denotes the slope of the set of data points. For sequence only alignments, this dependence of the apparent *λ *on sequence length is due to the "edge effect", which arises because the length of the longest island, and hence its associated score, is limited by the lengths of the sequences [21]. Thus, if the variance in slopes for profile-profile methods seen in Figure 4 is also due to the edge effect, one would expect to observe larger difference in slopes for methods that generate longer alignments. Indeed, our analysis of alignments generated by four methods in our study demonstrates that the variance in *λ *for small and large comparisons seen in Figure 4 scales proportionally with average alignment length generated by each method (30 for *WeihgtedLogOdds*, 47 for *CrossProduct*, 37 for *JensenShannon*, and 35 for *Multinomial*).

**Figure 5:**
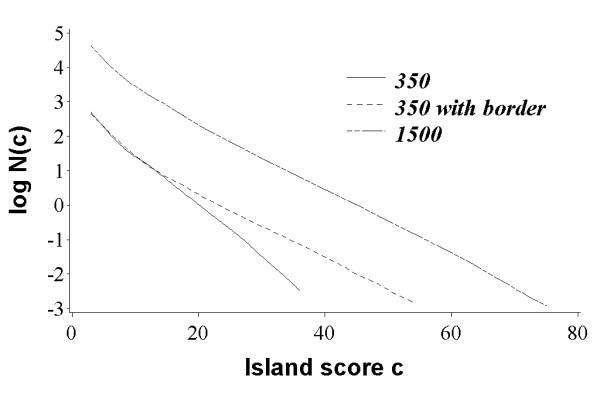
**The edge effect correction**. Semi-log plot of the observed and expected number of islands with score ≥ *c*. The dashed line represents the distribution obtained by surrounding the lattice of shuffled scores by a border of width 50 and counting only the islands anchored within the central 350 × 350 area.

The "edge effect" may be corrected for by allowing the islands to extend beyond the ends of the sequences [21]. For sequence only methods, this is done by embedding each *n *× *n *comparison within a lager comparison with a border of length *b *and then collecting only the islands anchored within the central *n *× *n *region [21]. We tested a similar technique for computing profile-pair specific asymptotic parameters from small size comparisons. We note that our procedure is slightly different from the procedure described in [21] because it treats the boundary and the central region separately. More specifically, to account for compositional bias in the profiles, only the scores in the central *n *× *n *square are shuffled and the boundary is filled in with scores chosen at random from the central region. Figure 5 shows the asymptotic distribution of island scores obtained from a comparison of size 350 × 350 surrounded by a border of size 50.

To assess the accuracy of the island method, we (like Altschul *et al*. [21]) compute, for each island score cutoff *c*, the estimates of *λ *and *K *using equations 3 and 4. Table 1 gives the island estimates of *λ *and *K *for a single pair of random profiles of lengths 1,500 using the *WeightedLogOdds *scoring function. Similar results were obtained with the other three scoring functions (data not shown).

**Table 1:** Island estimates of *λ *and *K*

*c*	*R*_*c*_		SE	
20	18942541	0.1924	0.02%	0.0395
21	15451236	0.1899	0.03%	0.0371
22	12673131	0.1882	0.03%	0.0354
23	10416563	0.1866	0.03%	0.0339
24	8557899	0.1846	0.03%	0.0320
25	7041327	0.1825	0.04%	0.0300
26	5794787	0.1800	0.04%	0.0278
27	4796202	0.1782	0.05%	0.0262
28	3981201	0.1767	0.05%	0.0249
29	3312692	0.1753	0.05%	0.0238
30	2761460	0.1740	0.06%	0.0227
31	2307980	0.1730	0.07%	0.0219
32	1931516	0.1720	0.07%	0.0211
33	1618724	0.1712	0.08%	0.0204
34	1358277	0.1704	0.09%	0.0198
35	1141702	0.1697	0.09%	0.0193
36	960448	0.1692	0.10%	0.0188
37	809392	0.1688	0.11%	0.0185
38	681757	0.1683	0.12%	0.0181
39	575054	0.1679	0.13%	0.0179
40	484923	0.1675	0.14%	0.0175
41	409305	0.1671	0.16%	0.0172
42	345792	0.1668	0.17%	0.0169
43	292455	0.1666	0.18%	0.0168
44	247162	0.1663	0.20%	0.0166
45	209396	0.1664	0.22%	0.0167
46	177245	0.1664	0.24%	0.0166
47	149811	0.1661	0.26%	0.0163
48	127130	0.1664	0.28%	0.0166
49	107539	0.1663	0.30%	0.0165
50	91004	0.1661	0.33%	0.0164
51	77013	0.1660	0.36%	0.0163
52	65225	0.1660	0.39%	0.0163
53	55132	0.1656	0.43%	0.0159
54	46798	0.1659	0.46%	0.0162
55	39719	0.1663	0.50%	0.0166
56	33618	0.1662	0.55%	0.0165
57	28394	0.1657	0.59%	0.0160
58	24094	0.1660	0.64%	0.0163
59	20398	0.1659	0.70%	0.0161
60	17282	0.1659	0.76%	0.0162
61	14634	0.1659	0.83%	0.0162
62	12430	0.1663	0.90%	0.0166
63	10497	0.1659	0.98%	0.0161
64	8837	0.1647	1.06%	0.0149
65	7579	0.1667	1.15%	0.0172
66	6407	0.1665	1.25%	0.0169
67	5416	0.1662	1.36%	0.0165
68	4591	0.1662	1.48%	0.0165
69	3892	0.1663	1.60%	0.0167
70	3280	0.1654	1.75%	0.0156
71	2786	0.1658	1.89%	0.0160
72	2367	0.1663	2.06%	0.0167
73	1985	0.1645	2.24%	0.0145
74	1695	0.1656	2.43%	0.0158

The estimates of *λ *and *K *and the standard error (SE) in  for a pair of random profiles obtained with the island method (equations 3 and 4) from 10,000 comparisons of size 1500 × 1500 using the *WeightedLogOdds *scoring scheme. The parameters estimated with the direct method are  = 0.16636,  = 0.01677.

**Figure 6:**
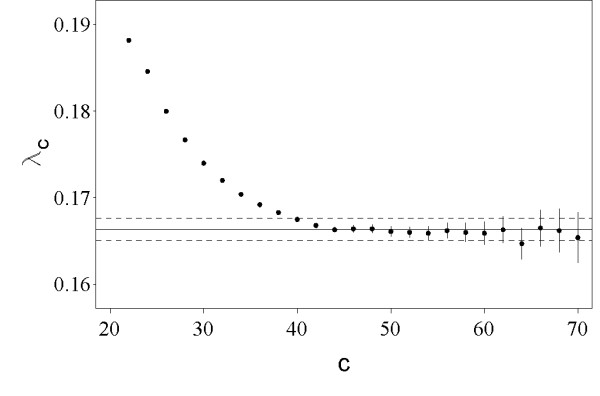
**Island method estimates of *λ***. The values of  from Table 1. The solid horizontal line corresponds to direct method estimate of *λ *obtained from 10,000 globally optimal local alignments between profile shuffles. The standard errors are shown as vertical lines for the island method and the dashed horizontal lines for the direct method.

To better illustrate the dependence of the island estimate of *λ *on the cutoff value *c*, we plot the values  from Table 1 in Figure 6. As seen in Figure 6, the value of  decreases with increasing island cutoff score *c*, until it reaches the value of 0.166 (direct method estimate of *λ*) at *c *= 44 and then randomly oscillates around this point.

### Speed vs. accuracy

There are two types of errors that can occur when computing the statistical parameters using random simulations. The first error, called "bias", represents the difference between the estimated and "true" statistical parameters. The second error is the standard error, which, unlike the bias, can be controlled by the number of data points used in parameter estimation. More specifically, the standard error in  is 1/ for the island method and 0.78/ for the direct method [21], where *R *denotes the number of data points, i.e. the number of island scores above the cutoff and the number of optimal alignment scores, respectively.

Both direct and island method suffer from bias in the estimates of the statistical parameters. As seen in Figure 6, the bias of the island method is closely related to the island cutoff score. Similarly, the direct method tends to overestimate *λ *due to the nonexistence of an optimal alignment score threshold. The maximum likelihood estimates of distribution parameters obtained with the direct method most strongly depend on the low scoring data points, because of the steep decrease of the left tail of the extreme value distribution. Therefore, the extent of bias for the direct method is proportional to the fraction of low scoring optimal alignments used for parameter estimation.

We note that the biases of the direct and island method can be computed (and compared) for local alignments of single sequences, due to availability of experimentally verified "best estimate" of the asymptotic *λ *[21]. Using the "best estimate" of *λ *as the reference point, Altschul and co-workers were able to find a threshold island score that eliminates all cutoff-based bias for large size comparisons of random sequences. By considering only the islands with peak scores over the threshold, they computed accurate, sequence length specific parameter estimates of *λ*, and used these estimates as gold standards to assess the extent of bias for both methods [21].

Unfortunately, it would be difficult to perform a similar experiment in our setting because of the dependence of statistical parameters on profiles' composition and because of the computational complexity of profile-profile methods. Thus, instead of comparing the bias side-by-side, we focus our attention on measuring the difference between the island and direct method estimates of *λ *and on comparing the computational efficiencies of two methods.

The speed advantage of the island method is due to its ability to generate multiple data points in a single comparison of two shuffled profiles. However, the average number of islands per pair of shuffled profiles does not directly translate into the speed advantage of the island method. First, for the same standard error in , the island method needs to generate 64% more data points than the direct method. Second, a single comparison of two profiles with the island method is computationally more expensive than the same comparison with the direct method, since the island method needs to keep track of the islands and their peak scores. Our implementation of the dynamic programming engine for the island method is ~1.5 times slower than the procedure that only returns an optimal alignment score. Taking those two factors into consideration, the total speed advantage of the island method is about *A*_*c*_/2.4, where *A*_*c *_denotes the average number of island with peak scores ≥ *c *collected in a single comparison of two shuffled profiles. We note that our results are identical to previously reported results for sequence-sequence alignments [21].

**Figure 7:**
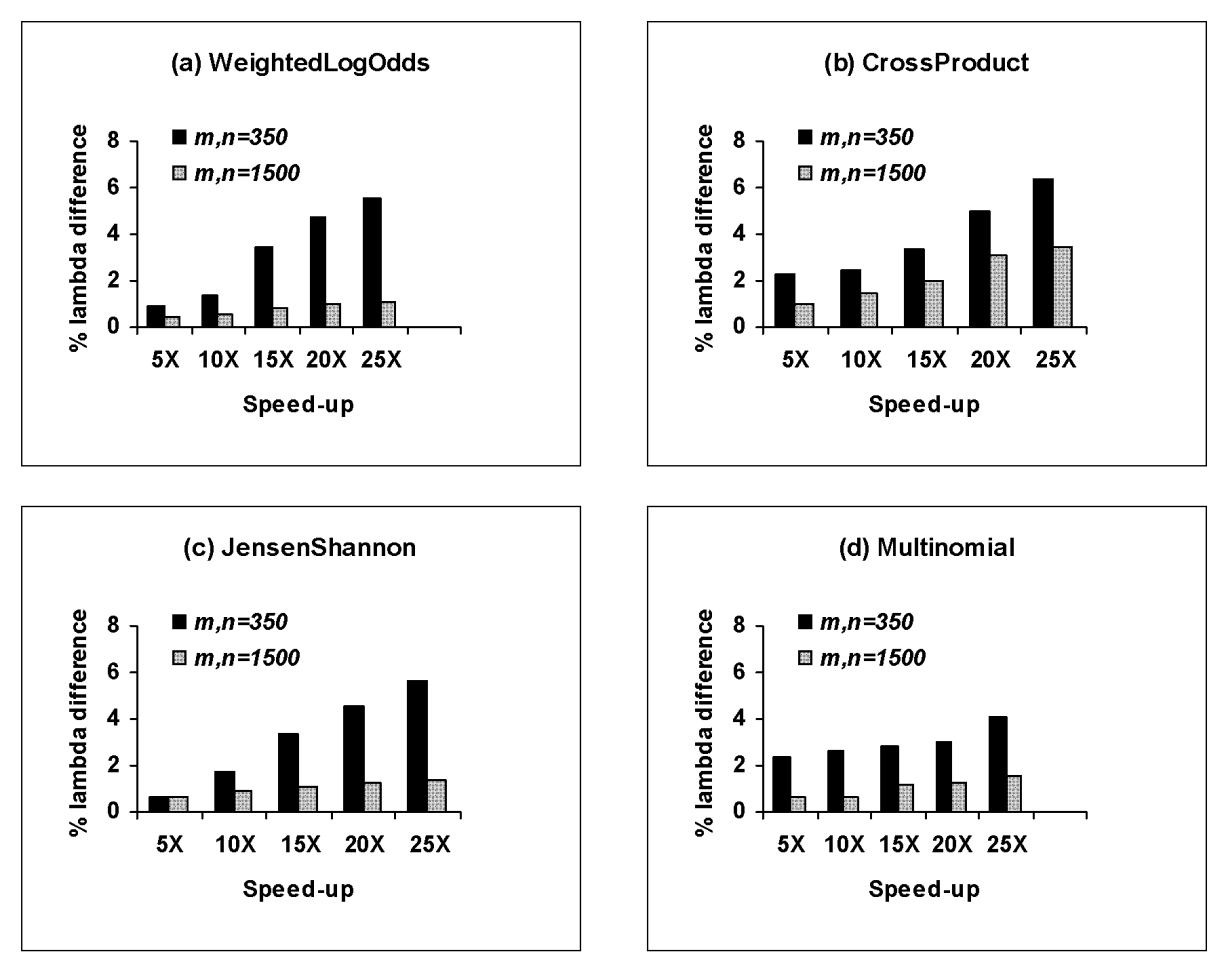
**Speed-up vs. the difference in the estimates of *λ***. The speed advantage of the island method and the deviation of the island estimates of *λ *from the parameters obtained by the direct method. The island scores and the optimal alignment scores were collected from 10,000 comparisons between the shuffles of random profiles. The results are averaged over 100 pairs of random profiles.

We emphasize that the speed advantage of island method also depends on the scoring scheme used in a profile-profile method. Figure 7 shows the relationship between the speed-advantage of the island method and the discrepancy in estimates of *λ *obtained with two methods. As seen in Figure 7, for the same speed-up, the difference in the estimates of *λ *obtained by two methods is smaller for large size comparisons. This is expected, because, for two equal size collections of top scoring islands, the average island score for a large size comparison exceeds the average island score for small size comparison, resulting in overall more accurate parameter estimates.

To compute the actual running times of two methods, we tested both programs on an Intel Xeon 2.13 GHz CPU computer with 4 GB of RAM. Table 2 gives the relationship between the running time of the island method and percent deviation of the island estimates of *λ *from the estimates obtained with the direct method (using direct method estimates as reference points). As seen in Table 2, for a typical comparison of size 350 × 350, the island method using the *JensenShannon *scoring function needs about 4 seconds to obtain an estimate of *λ *within 4% of the direct method estimate (standard error 0.78%). To achieve the same standard error in , the direct method requires ~1.3 minutes, corresponding to a 20-fold speed advantage of the island method. When compared to the direct method, the efficiency of the island method further increases with increasing lengths of the profiles. For instance, for the same 4% difference in the estimates of *λ *and comparisons of size 1500 × 1500, the island method is 100 times faster than the direct method (16 seconds vs. ~1/2 hour). For 2% difference in *λ*, the island method is 10 times faster for comparisons of size 350 × 350 and 30 times faster for comparisons of size 1500 × 1500. We note that increased computational efficiency on large profiles makes the island method particularly useful, since using the direct method to compute the parameters "on the fly" for large size comparisons would be computationally prohibitive.

**Table 2:** Running time of the island method and the deviation in *λ*

	***m, n *= 350**			***m, n *= 1500**		
**Method**	**2 *s***	**4 *s***	**8 *s***	**16 *s***	**32 *s***	**64 *s***

*WeightedLogOdds*	7%	5%	1%	4%	2%	1%
*CrossProduct*	10%	5%	2%	14%	7%	4%
*JensenShannon*	8%	4%	2%	4%	3%	2%
*Multinomial*	6%	3%	3%	5%	2%	1%

The actual running time of the island method and the (percent) difference in the island estimates of *λ *from the values obtained with the direct method. The results are averaged over 100 pairs of random profiles. The standard error in each estimate of *λ *for both methods is 0.78%. The running time of the direct method is ~1.3 minutes for comparisons of size 350 × 350 and ~30 minutes for comparisons of size 1500 × 1500.

We emphasize that, by using the direct method estimates as reference points, we do not argue that these estimates are more accurate than the estimates obtained with the island method. In fact, the results of a similar analysis for sequence-only methods [21] suggest that, for comparisons of size ~350 × 350, the bias of the direct method would be about three times larger than the bias of the island method, for the same standard error in .

Previous studies of the island statistics for sequence-sequence alignments addressed the speed-accuracy tradeoff by optimizing the island score cutoff *c*. For the BLOSUM62 matrix and gap opening and extension penalties of 11 and 1, respectively, the cutoff value of *c *= 28 was found appropriate [21]. Olsen and co-workers suggested the cutoff value of *c *= 1.3·*max*{*s*_*ab*_}, where *s*_*ab *_is the score for matching amino acid letters *a *and *b*, specified in the substitution matrix [22].

A slightly different interpretation of the results in Table 2 suggests an alternative approach to controlling speed and accuracy tradeoff for an arbitrary profile-profile scoring scheme and a range of profile lengths. For example, for a pair of profiles of lengths 350, the *JensenShannon *scoring scheme, and the standard error of 0.78%, the island estimate of *λ *that is within 4% of the direct method estimate of *λ *can be obtained by running the island method for ~4 seconds and computing *λ *using the top scoring 16,437 islands (this number of islands yields standard error in  of 0.78%).

We used our in-house computer cluster to directly compare the performance of the island and the direct method in identifying the relationships between the sequences in the Lindahl test set [42]. The Lindahl test set is composed of 1310 pairs of proteins classified in three groups according to SCOP[17] hierarchy. The accuracy of an alignment method in the Lindahl benchmark is defined as its ability to place a correct member of the SCOP group (family, superfamily, and fold) on the top of its ranked list. The results of our test, presented in Table 3, show no significant difference in fold recognition sensitivity between the two methods.

**Table 3:** Lindahl benchmark

	**Top 1**	**Top 5**
*Fold*	+1.2%	+0.9%
*Superfamily*	-0.9%	-0.5%
*Family*	-0.7%	+1.1%

The performance of the island method in the Lindahl benchmark for fold recognition. We show the difference in the percentage of the relationships identified by the island and the direct method (% island – % direct) in the first and the top five ranks, at each *SCOP *level (family, superfamily and fold). In this analysis we used the *Multinomial *scoring system. Similar results were obtained with the other three scoring schemes (data not shown).

## Conclusion

By utilizing the information present in protein families, profile-profile alignment algorithms are often able to detect extremely week relationships between protein sequences, as evidenced by the large scale benchmarking experiments such as CASP [43], CAFASP [44], and LiveBench [45]. However, estimating the score statistics for profile-profile alignments is a challenging problem. The background distribution of profile-profile alignment scores is constrained by profiles' composition and hence the distribution parameters must be estimated independently, for each given pair of profiles.

We study the applicability of the well known "island method" to profile-profile score normalization. In the island method, the statistical parameters are computed based upon the top scoring islands that can be collected using a simple modification of the Smith-Waterman algorithm. Since multiple high scoring islands can be extracted from a single path graph, the island method has a distinct speed advantage over the direct method. For some widely used profile-profile scoring schemes, the speed advantage of the island method exceeds an order of magnitude for comparable accuracy in parameter estimates. For larger profiles, a significant speed advantage of the island statistics comes with almost perfect accuracy. This is important, since using the direct method as the only other alternative to compute the parameters "on the fly" for large size comparisons is computationally prohibitive.

## Appendix

The *JensenShannon *score [18] between probability distributions *q*^1 ^and *q*^2 ^is defined as

(5)

where *J *= *D*^*JS *^(*q*^1^, *q*^2^) is the *Jenson-Shannon *divergence between *q*^1 ^and *q*^2 ^and *S *= *D*^*JS *^(*r*, *b*) is the *Jenson-Shannon *divergence between the "most likely common source distribution" *r *for *q*^1 ^and *q*^2 ^and the "overall" distribution of 20 amino acid letters *b*. The distribution *r *is defined as

(6)

The *Jenson-Shannon *divergence is given by

(7)

where *D*^*KL *^is the Kullback-Leibler divergence

(8)

The *CrossProduct *scoring function [39] multiplies the products of the amino-acid target frequencies by the corresponding elements *s*_*ab *_of the BLOSUM62 substitution matrix

(9)

The *WeightedLogOdds *[14] and the *Multinomial *[20] scoring functions use the effective amino-acid counts when scoring a pair of profile positions. More specifically, the score for matching *q*^1 ^and *q*^2 ^is given as

(10)

where  and  are the "effective counts" for the amino acid *k *observed at two profiles' columns and *b*_*k *_is the background probability of *k*. In the *WeightedLogOdds *function, the parameters *c*_1 _and *c*_2 _are set to

(11)

(12)

In the *Multinomial *scoring function, both *c*_1 _and *c*_2 _are set to 1.
